# Cholecystokinin A Receptor Knockdown Diminishes Colon Cancer Cell Invasive Potential via Modulation of Integrin/FAK, EMT, and uPA/uPAR/MMP2 Axis

**DOI:** 10.32604/or.2026.074231

**Published:** 2026-03-23

**Authors:** Chun-Shiang Lin, Ta-Wen Hsu, Hsiang-Lin Lee, Shao-Hsuan Kao

**Affiliations:** 1 Institute of Medicine, College of Medicine, Chung Shan Medical University, Taichung, Taiwan; 2 Precision Medicine Center, Chung Shan Medical University Hospital, Taichung, Taiwan; 3 Division of Colorectal Surgery, Buddhist Tzu Chi Medical Foundation, Dalin Tzu Chi Hospital, Chiayi, Taiwan; 4 School of Medicine, Tzu Chi University, Hualien, Taiwan; 5 School of Medicine, Chung Shan Medical University, Taichung, Taiwan; 6 Department of Surgery, Chung Shan Medical University Hospital, Taichung, Taiwan; 7 Department of Medical Research, Chung Shan Medical University Hospital, Taichung, Taiwan

**Keywords:** Cholecystokinin A receptor (CCKAR), colon cancer, invasiveness, integrin, epithelial-mesenchymal transition (EMT)

## Abstract

**Objectives:**

Cholecystokinin A receptor (CCKAR) has been linked to poor prognosis in colon cancer patients, but the role of CCKAR in colon cancer cell invasiveness and the underlying mechanisms remain elusive. This study aimed to explore the effect of CCKAR on the invasive potential of colon cancer cells.

**Methods:**

Different human colon cancer cell lines were used. Gene expression was evaluated by reverse transcription polymerase chain reaction (RT-PCR) and quantitative real-time RT-PCR (qPCR), while protein expression and phosphorylation were assessed by Western blotting. Cell motility and invasiveness were examined through wound healing and invasion assays, respectively.

**Results:**

Our results showed that CCKAR expression levels varied across colon cancer cell lines, with DLD-1 and LoVo cells showing high expression. Knockdown of CCKAR significantly impaired the cell motility and invasiveness of DLD-1 and LoVo cells, downregulated integrin β3 expression, and diminished the phosphorylation levels of focal adhesion kinase (FAK), Src, and paxillin. In addition, CCKAR knockdown modulated epithelial-mesenchymal transition (EMT) markers ZO-1, E-cadherin, and vimentin and reduced urokinase-type plasminogen activator (uPA), uPA receptor (uPAR), Rho GTPase cell division control protein 42 (CDC42) and RhoA, and matrix metalloproteinase-2 (MMP-2).

**Conclusions:**

These findings indicate that CCKAR knockdown impairs the invasiveness of colon cancer cells, which may be attributed to modulating integrin/FAK/Rho GTPases, EMT markers, and the uPA/uPAR axis. It suggests that targeting CCKAR may represent a potential therapeutic strategy for colon cancer treatment.

## 1 Introduction

Colorectal cancer (CRC) ranks as the third most frequently diagnosed cancer globally and represents the second leading cause of cancer mortality worldwide [1]. Although significant progress has been made in screening protocols and treatment modalities, patient outcomes remain suboptimal, especially among individuals presenting with advanced metastatic disease [2]. Metastasis to distant anatomical sites largely accounts for the high mortality rates in colorectal malignancies [3]. Consequently, understanding the fundamental molecular pathways that drive invasive behavior in colorectal cancer represents a critical research priority for developing targeted therapeutic strategies and enhancing clinical outcomes.

The cholecystokinin (CCK) receptor family is classified as class-A group of sevenfold transmembrane G protein-coupled receptors. It includes the CCK-A receptor (CCKAR) and the CCK-B receptor (CCKBR) [4]. The CCKAR is a G protein-coupled receptor that governs a number of physiological functions, including stomach acid secretion, pancreatic enzyme release, and satiety [5]. CCKAR is primarily localized in the gastrointestinal tract [6], the vagal sensory neurons [7], and specific regions of the brain [8]. Beyond its expression in healthy tissues, CCKAR is also upregulated and associated with poor prognosis in various malignancies, including lung, colon, and gallbladder tumors [9,10]. Moreover, CCK enhances the proliferation and survival of tumor cells; hence, blocking the CCK receptor with its antagonists lowers proliferation and induces death of tumor cells, suppressing tumor growth *in vitro* and *in vivo* [11–13]. However, the role of CCKAR in tumor invasiveness remains unclear.

Integrins function as heterodimeric transmembrane glycoproteins that facilitate interaction between cells and the extracellular matrix, thereby initiating complex signaling networks that regulate cellular migration, survival, and proliferation [14]. Dysregulated integrin expression and altered signaling patterns are frequently observed across diverse malignancies and correlate strongly with increased metastatic capacity [15,16]. Upon integrin clustering at adhesion sites, focal adhesion kinase (FAK) is recruited and activated, establishing itself as a pivotal signaling hub that coordinates multiple pathways controlling cancer cell motility, invasiveness, and metastatic spread [16]. Through its interaction with downstream targets, including Src family kinases and the adaptor protein paxillin, FAK orchestrates cytoskeletal reorganization, focal adhesion dynamics, and the production of matrix metalloproteinases, collectively promoting enhanced cancer cell migration and tissue invasion capabilities [17].

The Rho GTPase family comprises small guanosine triphosphatases that orchestrate diverse cellular mechanisms encompassing cytoskeletal remodeling, morphological plasticity, cellular polarity, motile behavior, vesicular transport, cell cycle regulation, apoptotic resistance, and developmental programs [18]. Given their frequent deregulation in neoplastic contexts [19], Rho GTPases represent compelling molecular targets for anticancer therapeutic development [20].

The matrix metalloproteinase (MMP) family includes calcium-dependent zinc-containing proteolytic enzymes crucial for extracellular matrix degradation, tissue maintenance, and the pathogenesis of diseases, including cancer invasion and metastasis [21]. Among MMPs, MMP2 has been identified as a critical enzyme in oncology due to its capacity to cleave type IV collagen within basement membranes, creating pathways for cancer cell infiltration and distant metastasis [22].

A central mechanism driving the metastatic cascade is the Epithelial-Mesenchymal Transition (EMT) [23]. During this biochemical process, epithelial cells lose their characteristic polarity and cell-cell adhesion, subsequently acquiring a mesenchymal phenotype characterized by increased migratory capacity and invasiveness. In colorectal cancer, EMT is typically marked by the downregulation of epithelial markers such as E-cadherin and ZO-1, alongside the upregulation of mesenchymal markers like vimentin [23]. Importantly, EMT is not an isolated event, but is closely linked to the remodeling of the cytoskeleton and extracellular matrix, and is usually regulated by integrin signaling and expression of Rho GTPases [24]. Understanding how specific receptors like CCKAR influence this transition is therefore crucial for identifying drivers of tumor progression.

Based on the established roles of cholecystokinin receptors in cellular signaling, we hypothesize that CCKAR promotes colon cancer invasiveness by modulating a coordinated network involving integrin/FAK, EMT markers, Rho GTPases, urokinase-type plasminogen activator (uPA)/uPA receptor (uPAR), and MMP2 pathways. This study aims to test this hypothesis by characterizing the molecular consequences of CCKAR knockdown and its impact on the metastatic potential of colon cancer cells.

## 2 Materials and Methods

### 2.1 Reagents

All the unspecified chemicals and reagents were obtained from Sigma-Aldrich (St. Louis, MO, USA). Devazepide, CCKAR antagonist, was purchased from (HY-106301, MedChemExpress, New York, NY, USA). For Western blotting, antibodies specificically recognized human target proteins and their details were listed in supplementary Table S1. Peroxidase-conjugated antibodies to mouse (sc-2005) or rabbit IgG (sc-2004) were acquired from Santa Cruz Biotechnology (Santa Cruz, CA, USA).

### 2.2 Culture for Colon Cancer Cell Lines

Six human colon cancer cell lines, C2BBe1 (#60182, clone of Caco-2), DLD-1 (#60132), LoVo, (#60148), SW480 (#60249), and SW620 (#60343) were obtained from the Bioresource Collection and Research Center (Hsinchu, Taiwan). HT-29 was purchased from (GRL-CLC016, Asia Bioscience Co. ltd., Taipei, Taiwan). Cells were verified to be free of mycoplasma contamination using PCR Mycoplasma Detection kit (#G238, Omics Bio Inc., New Taipei City, Taiwan). Cell morphology was monitored regularly, and all assays were conducted within low passage numbers to maintain phenotypic consistency. Cells were maintained in Dulbecco’s Modified Eagle’s Medium (#31600034, DMEM, Gibco, Grand Island, NY, USA, for C2BBe1, HT-29), Ham’s F-12 medium (#21700075, Gibco, for LoVo), L-15 culture medium (#41300039, Gibco, for SW480, SW620), or RPMI-1640 (#11875093, Gibco, for DLD-1) containing heat-inactivated 10% fetal bovine serum (FBS; #04-121-1A, Biological Industries, Cromwell, CT, USA) and incubated in a humidified incubator with 5% CO_2_ at 37°C. Cells grown to 80% confluency were harvested by incubation with trypsin-EDTA for the subsequent treatments and analyses. All the cell lines were purchased from the Bioresource Collection and Research Center, Hsinchu, Taiwan, and their genetic origin was confirmed by the supplier through STR-PCR analysis.

### 2.3 RT-PCR and Quantitative Real-Time PCR for mRNA Expression Assessment

Total RNA (100 ng) was isolated using TRIzol reagent (#15596026, Thermo Fisher Scientific, Waltham, MA, USA) and reverse-transcribed into cDNA using the ReverTra Ace qPCR RT Kit (#FSQ-101, TOYOBO, Osaka, Japan) per the manufacturer’s protocol. Target mRNA levels, specified by the primers in Table 1, were normalized to GAPDH expression. Relative quantification was calculated using the 2^−ΔΔCt^ method, following established procedures [25]. Briefly, PCR amplifications were performed in triplicate using 50 μL reaction volumes qPCR master mix (#FSQ-201, TOYOBO) containing 2.5 μL of cDNA and 200 nM of each primer.. The amplification protocol included an initial 5-min denaturation at 94°C, followed by 35 cycles of 94°C (30 s), 55°C (30 s), and 72°C (30 s), with a final extension at 72°C for 7 min. Both RT-PCR and qPCR analyses were performed in triplicate using three independent biological replicates.

**Table 1:** Primers used in qPCR for mRNA expression assessment.

Gene	Forward (5^′^ to 3^′^)	Reverse (5^′^ to 3^′^)
CCKAR	CGC TTTTCTGCTTGGATCAGCC	GCTTGTTCCGAATCA GCACGGT
MMP2	AGCGAGTGGATGCCGCCTTTAA	CATTCCAGGCATCTGCGATGAG
ITGAV	AGGAGAAGGTGCCTACGAAGCT	GCACAGGAAAGTCTTGCTAAGGC
ITGB3	CATGGATTCCAGCAATGTCCTCC	TTGAGGCAGGTGGCATTGAAGG
FAK	GCCTTATGACGAAATGCTGGGC	CCTGTCTTCTGGACTCCATCCT
SRC	CTGCTTTGGCGAGGTGTGGATG	CCACAGCATACAACTGCACCAG
Paxillin	CTGATGGCTTCGCTGTCGGATT	GCTTGTTCAGGTCAGACTGCAG
ZO-1 (TJP1)	GTCCAGAATCTCGGAAAAGTGCC	CTTTCAGCGCACCATACCAACC
E-cadherin	GAGAACGCATTGCCACATACA	ACCTTCCATGACAGACCCCTTAA
Vimentin	AGGCAAAGCAGGAGTCCACTGA	ATCTGGCGTTCCAGGGACTCAT
RhoA	TCTGTCCCAACGTGCCCATCAT	CTGCCTTCTTCAGGTTTCACCG
CDC42	TGACAGATTACGACCGCTGAGTT	GGAGTCTTTGGACAGTGGTGAG
GAPDH	GAGTCAACGGATTTGGTCGT’	TTGATTTTGGAGGGATCTCG

### 2.4 Gene Knockdown by shRNA Lentivirus Infection

Stable CCKAR expression knockdown was achieved by using shRNA. Briefly, complementary oligonucleotides were ligated into pLKO.1-puro, and lentiviral particles were produced by co-transfecting HEK293T packaging cells with the shRNA-expression vector and packaging plasmids psPAX2 (#12260, addgene, Watertown, MA, USA) using Lipofectamine 3000 (Invitrogen, Carlsbad, CA, USA). Cells grown to 80% confluency were infected with lentiviral particles carrying negative control shRNA constructs (sh-NC, target sequence UUCUCCGAACGUGUCACGUTT) or CCKAR shRNA constructs (sh-CCKAR, GAATTCCCACAACGCCACTCA)(National RNA Interference Core Facility at the Institute of Molecular Biology, Academia Sinica, Taipei, Taiwan) at a specific multiplicity of infection (MOI) of 10 for 48 h according to the instruction. DLD-1 and LoVo cells with stable CCKAR knockdown were acquired by using a two-week puromycin selection procedure and the knockdown efficiency was evaluated by qPCR and Western blotting.

### 2.5 Western Blotting

For crude protein extraction, cells were washed with PBS, harvested by trypsin-EDTA incubation. After washed with PBS, the cells were lysed with a RIPA buffer (#RP-05-100, Visual Protein, Taipei, Taiwan). Protein concentration was assessed by the Bradford method according to the manufacturer’s instructions (#5000002, Bio-Rad Laboratories, Inc., Hercules, CA, USA). Equal amount of protein (20 μg) was electrophoresed in 12.5% SDS-polyacrylamide gel and transferred onto PVDF membranes. After being blocked with 2% BSA/PBS for 1 h at room temperature (RT), the transferred PVDF was incubated with 1:1500-diluted primary antibodies for 2 h at RT, then incubated with 1:2000-diluted HRP-conjugated secondary antibodies for 1 h at RT. Signal development was carried out using a chemiluminescent substrate (#LF01-500, Visual Protein), and the resulting chemiluminescent signals were acquired and relatively quantitated by a LAS-4000 mini Luminescent Image Analyzer (GE Healthcare Bio-Sciences, Uppsala, Sweden). Band intensities for target proteins and their phosphorylated forms were normalized to β-actin as an internal loading control. Relative protein expression was calculated as the ratio of the target protein’s signal intensity to that of β-actin. All experiments were performed in duplicate.

### 2.6 Assessment of Cell Motility

Cell motility potential was assessed by wound healing and invasion assays.

For the wound healing assay, 2 × 10^5^ cells per well were seeded into 12-well plates containing complete medium (10% FBS, Ham’s F-12 medium for LoVo and RPMI-160 for DLD-1), cultured at 37°C for 24 h to form cell monolayer, and made a scratch by a sterile 10 μL pipette tip. Then, the cells were incubated with serum-free medium (Ham’s F-12 medium for LoVo and RPMI-160 for DLD-1) for 24 h, and the scratch area at 0 and 24 h were photographed by Zeiss Axio Imager A2 microscope (Zeiss, NY, USA) with a 10× objective lens and quantified using the ImageJ software (version 1.54p, National Institutes of Health, Bethesda, USA).

For the invasion assay, 2 × 10^4^ cells were suspended in 0.5 mL medium containing 0.5% FBS and transferred into the upper chamber of transwell inserts (#3422, 8.0 μm pore size, Corning, Sigma-Aldrich) pre-coated with 100 μL Matrigel (20-fold dilution in PBS, CLS354234, Corning). The complete medium containing 10% FBS was added to the lower chamber. After 24 h of incubation, non-invading cells on the upper surface were gently removed with a cotton swab. The transmigrated cells on the lower surface were fixed, stained with Giemsa solution (#32884, Sigma-Aldrich), and photographed for cell number counting.

### 2.7 MMP-2 Production Assessment

The changes of secreted MMP-2 were assessed using an ELISA kit (#KHC3081, Thermo Scientific, Rockford, IL, USA) following the manufacturer’s instructions. Briefly, conditioned media were collected and clarified by centrifugation at 12,000× *g* for 15 min at 16°C. Aliquots of the supernatant (50 μL) were processed according to the manufacturer’s instructions, with a 1-h incubation at RT. Following color development, absorbance was measured at 450 nm using a photospectrometer (SpectraMax M5, Molecular Devices, Sunnyvale, CA, USA) operating in absorbance mode. Relative MMP-2 production was calculated as the ratio of optical density (OD) values between the sh-NC and sh-CCKAR groups. Results were derived from two independent experiments.

### 2.8 Statistical Analysis

Data are presented as the mean ± standard deviation (SD) of two or three independent experiments. Comparisons between two groups were performed using the paired Student’s *t*-test. Differences between groups were assessed using one-way analysis of variance (ANOVA) followed by the Tukey test for comparisons between more than two groups. A *p*-value ≤ 0.05 was considered statistically significant. All statistical analyses and graphs were performed using SigmaPlot software (version 10, Grafiti LLC, Palo Alto, CA, USA).

## 3 Results

### 3.1 Differential CCKAR Expression in Colon Cancer Cell Lines

**Figure 1:**
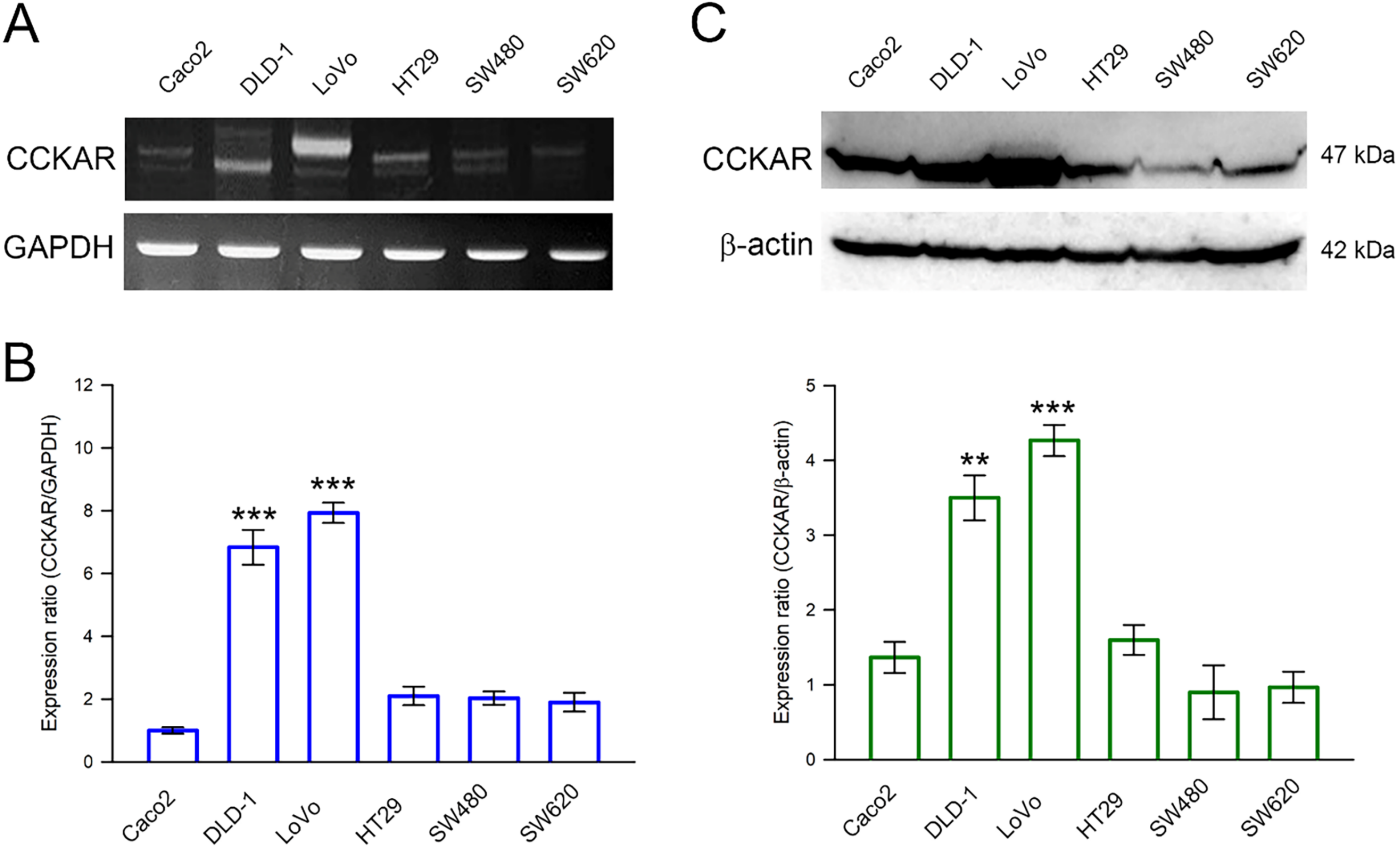
Expression profile of CCKAR in human colon cancer cells. Cells were collected and lysed for total RNA and crude protein extraction. The extracted RNA was reverse-transcribed, and the CCKAR mRNA expression level was determined by using (**A**) reverse transcription-polymerase chain reaction (RT-PCR) and (**B**) quantitative real-time RT-PCR (qPCR). (**C**) The extracted crude proteins were subjected to immunodetection of CCKAR using Western blotting. Semi-quantitation of signals was assessed by densitometric analysis. The expression of GAPDH and the signal of β-actin were used as an internal control for qPCR and Western blotting, respectively. ** and ***, *p* < 0.01 and *p* < 0.001 vs. control.

First, we detected the expression of CCKAR mRNA in six human colon cancer cell lines Caco2, DLD-1, LoVo, HT29, SW480, and SW620, using RT-PCR and qPCR. As shown in Fig. 1A,B, CCKAR mRNA was significantly higher in DLD-1 and LoVo cell lines compared with the other colon cancer cell lines. Consistently, western blot demonstrated higher protein expression levels of CCKAR in DLD-1 and LoVo cell lines, compared with those in Caco2, HT29, SW480, and SW620 cells (Fig. 1C). DLD-1 and LoVo cells displayed a higher expression level of CCKAR.

### 3.2 CCKAR Knockdown Impairs the Metastatic Potential of Colon Cancer Cells

**Figure 2:**
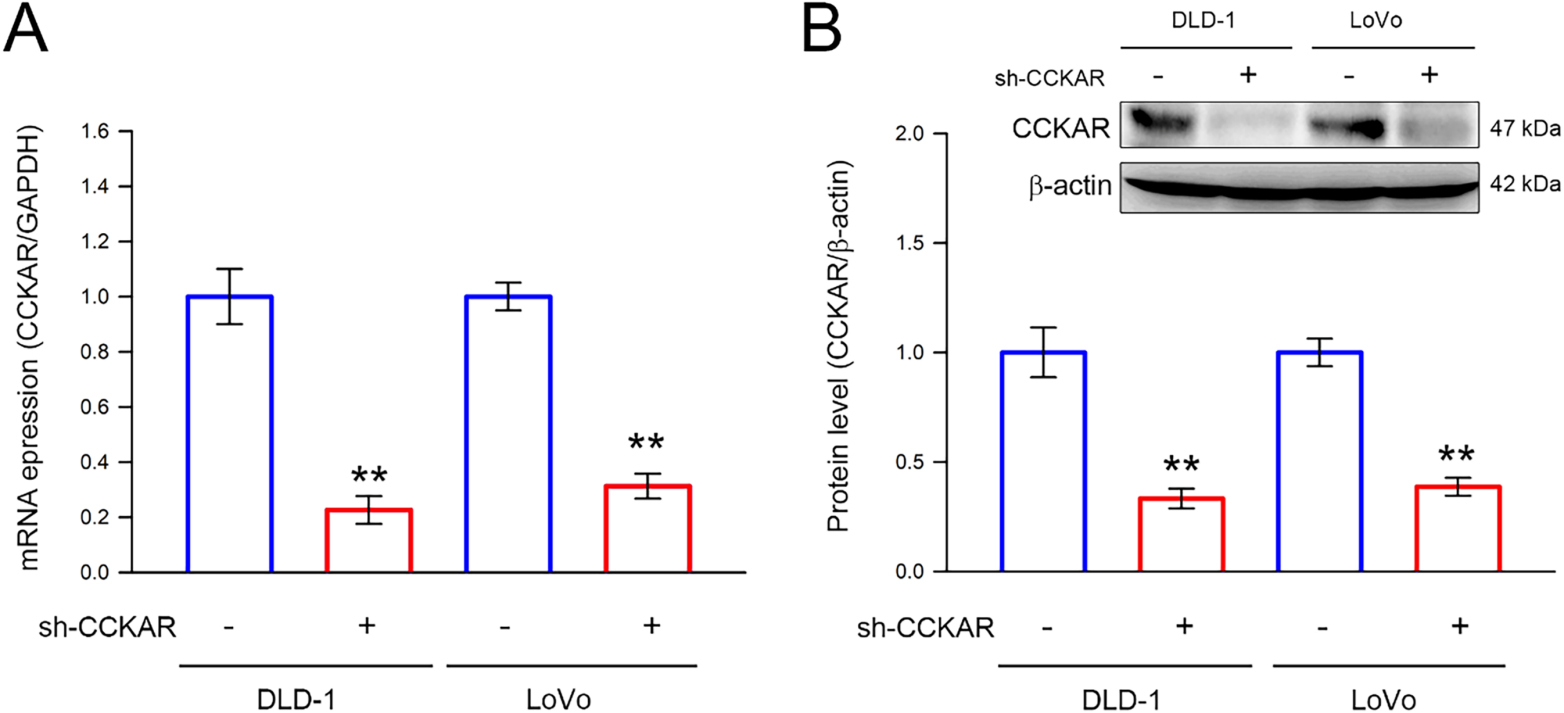
Knockdown of CCKAR in human colon cancer cells. DLD-1 and LoVo cells were transfected with specific shRNA lentiviral constructs against CCKAR (sh-CCKAR) or control lentiviral constructs (sh-NC). The transfected cells were subjected to (**A**) qPCR analysis for assessment of CCKAR mRNA expression level, or (**B**) immunodetection of CCKAR using Western blotting. Semi-quantitation of signals was assessed by densitometric analysis. The expression of GAPDH and the signal of β-actin was used as an internal control for qPCR and Western blotting, respectively. **, *p* < 0.01 vs. sh-NC.

**Figure 3:**
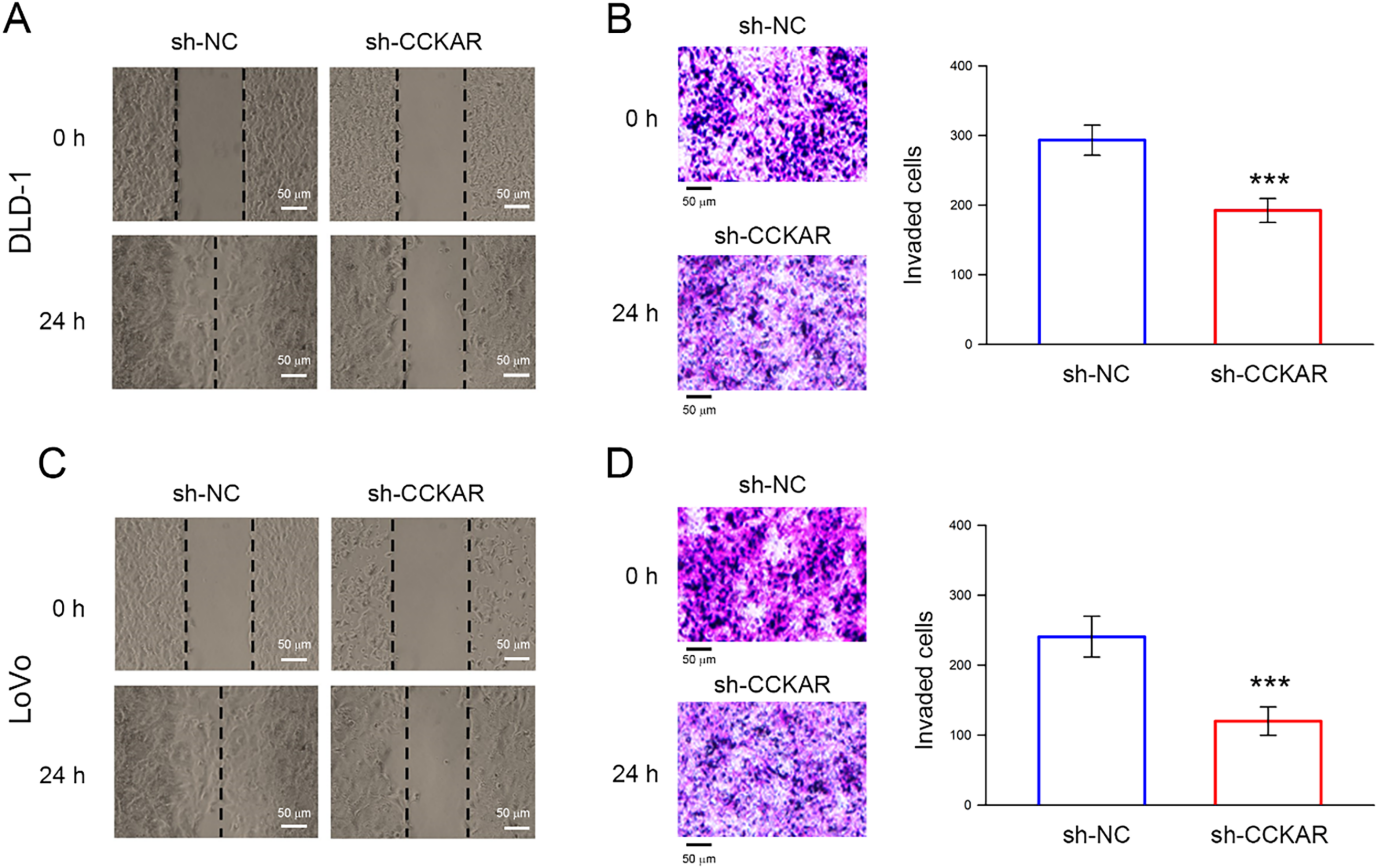
Knockdown of CCKAR impairs the cell migratory and invasive potential of human colon cancer cells. DLD-1 and LoVo cells were transfected with specific shRNA lentiviral constructs against CCKAR (sh-CCKAR) or control lentiviral constructs (sh-NC). (**A**) Wound healing assay for assessment of migratory potential of DLD-1, (**B**) Invasion assay for assessment of invasive potential of DLD-1, (**C**) Wound healing assay for assessment of migratory potential of LoVo, (**D**) Invasion assay for assessment of invasive potential of LoVo. Scale bar: 50 μm. ***, *p* < 0.001 vs. sh-NC.

To further explore the role of CCKAR in the invasiveness of colon cancer cells, stable CCKAR-knockdown DLD-1 and LoVo cells were established by transfection of shRNA against CCKAR (sh-CCKAR). Cells transfected with control shRNA (sh-NC) were used as control. In DLD-1 and LoVo cells, sh-CCKAR transfection resulted in a reduction of CCKAR mRNA to an average of 23% and 31% of control levels, respectively (Fig. 2A; *p* < 0.01). Consistently, in sh-CCKAR-transfected DLD-1 and LoVo cells, CCKAR protein levels were 38% and 40% of the levels observed in control cells, respectively (Fig. 2B; *p* < 0.01). Then, the CCKAR-knockdown cells were subjected to wound healing assay and invasion assay. As shown in Fig. 3A,B, CCKAR knockdown diminished the capability of cell migration and invasive potential of DLD-1 cells compared with control. Similarly, CCKAR knockdown also reduced the capability of cell migration and invasive potential of LoVo cells to an average of 63% and 48% of control levels, respectively (Fig. 3C,D; *p* < 0.001). Furthermore, there was no significant change in proliferation between cells transfected with sh-NC and sh-CCKAR (Supplementary Fig. S1).

### 3.3 CCKAR Knockdown Inhibits FAK/Src/Paxillin Signaling and Downregulates Expression of Integrin **β**3 and Paxillin in Colon Cancer Cells

**Figure 4:**
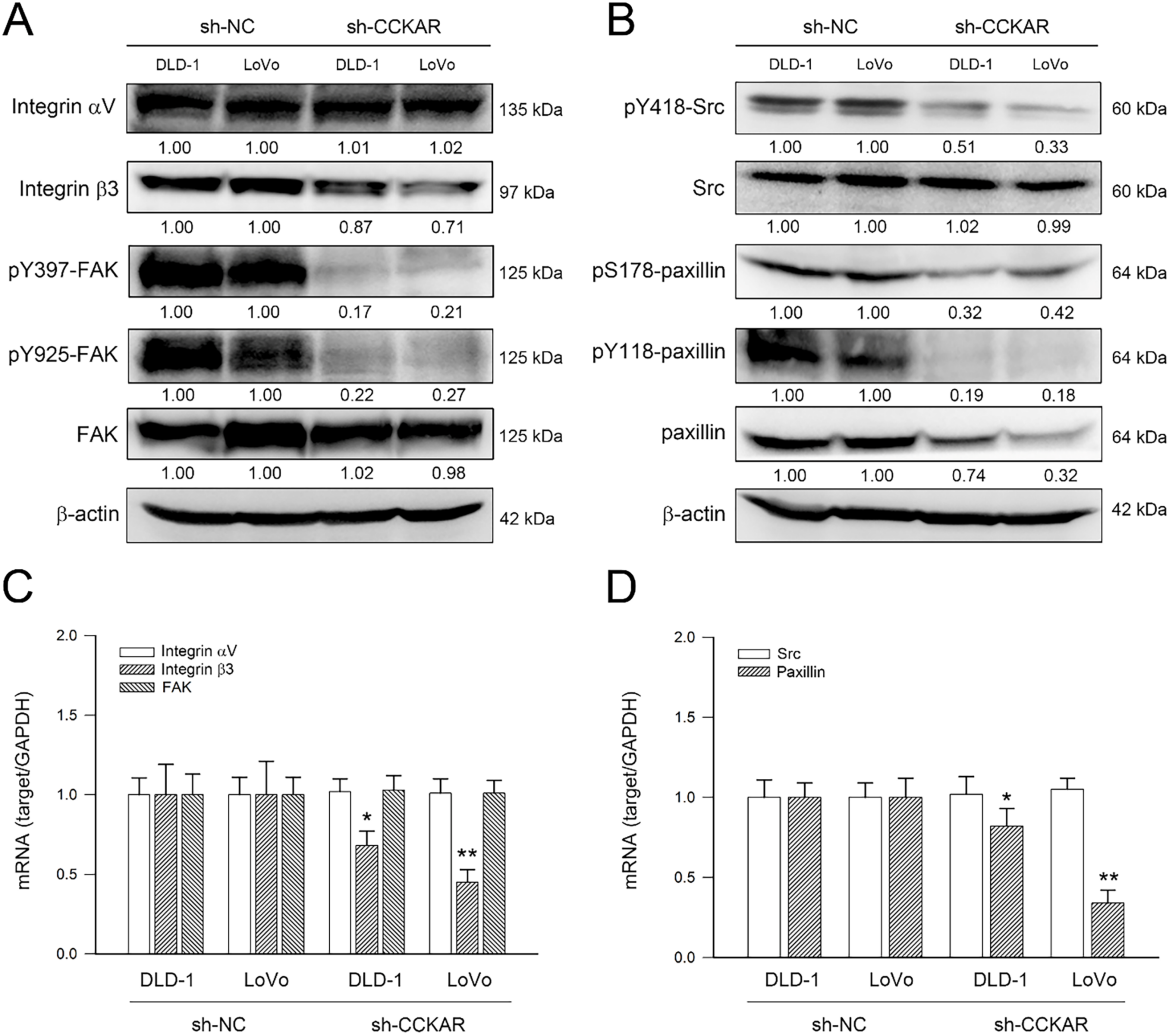
Knockdown of CCKAR reduces integrin β3 and paxillin expression and attenuates FAK/Src/paxillin signaling in human colon cancer cells. DLD-1 and LoVo cells were transfected with specific shRNA lentiviral constructs against CCKAR (sh-CCKAR) or control lentiviral constructs (sh-NC). The transfected cells were subjected to Western blot analysis for assessment of expression and phosphorylation of (**A**) integrin isoforms and FAK and (**B**) Src and paxillin or qPCR analysis for mRNA expression of (**C**) integrin αV/β3 and FAK and (**D**) SRC and paxillin. Semi-quantitation of signals was assessed by densitometric analysis. The expression of GAPDH and the signal of β-actin was used as an internal control for qPCR and Western blotting, respectively. * and **, *p* < 0.05 and *p* < 0.01 vs. sh-NC.

Integrins and their downstream signaling play important roles in the metastatic potential of colon cancer cells [17]. Therefore, whether CCKAR knockdown influenced integrin expressions and their downstream signaling was investigated. As shown in Fig. 4A, CCKAR knockdown reduced integrin β3 and FAK phosphorylation levels at Tyr397 and Tyr925. In addition, CCKAR knockdown declined Src phosphorylation at Tyr418 and paxillin phosphorylation at Ser178 and Tyr118 (Fig. 4B). Notably, CCKAR knockdown also reduced the protein level of paxillin; however, it had no significant effect on the protein level of integrin αV. In parallel to protein level, the impacts of CCKAR knockdown on gene transcription were evaluated. As shown in Fig. 4C,D, CCKAR knockdown similarly downregulated the mRNA expression of integrin β3 and paxillin to 67% and 43% of control in average (*p* < 0.05 vs. sh-NC) but not integrin αV, FAK, and Src.

### 3.4 CCKAR Knockdown Modulates Expression of EMT Makers, Rho GTPases, uPA/uPAR, and MMP2 in Colon Cancer Cells

**Figure 5:**
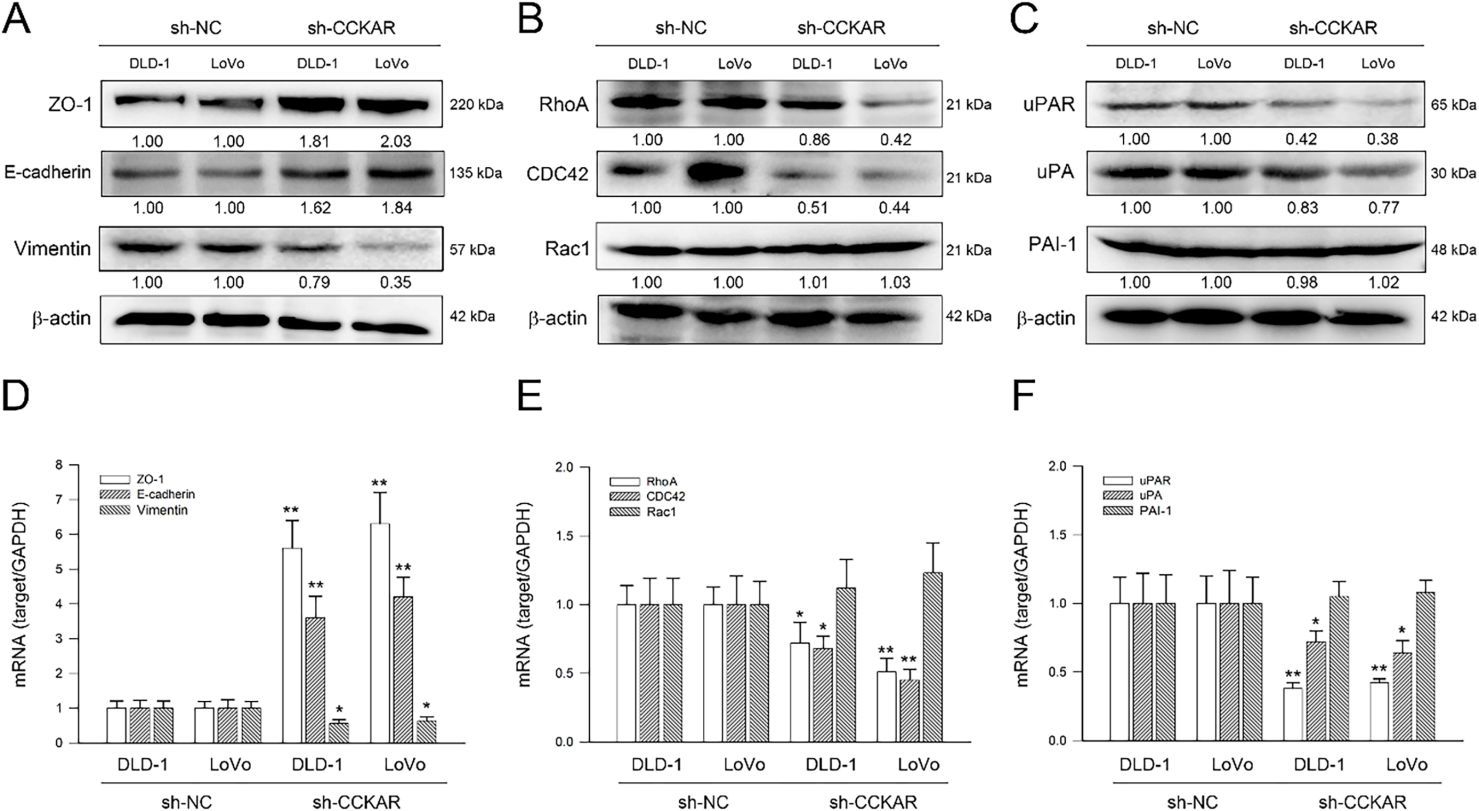
Knockdown of CCKAR regulates expression of EMT makers, Rho GTPases, and uPAR/uPA in human colon cancer cells. DLD-1 and LoVo cells were transfected with specific shRNA lentiviral constructs against CCKAR (sh-CCKAR) or control lentiviral constructs (sh-NC). The transfected cells were subjected to Western blot analysis for assessment of the protein level of (**A**) ZO-1, E-cadherin, and vimentin, (**B**) Rho A, CDC42, and Rac1, and (**C**) uPAR, uPA, and PAI-1 or qPCR analysis for mRNA expression of (**D**) ZO-1, E-cadherin, and vimentin, (**E**) Rho A, CDC42, and Rac1, and (**F**) uPAR, uPA, and PAI-1. Semi-quantitation of signals was assessed by densitometric analysis. The expression of GAPDH and the signal of β-actin were used as internal controls for qPCR and Western blotting, respectively. * and **, *p* < 0.05 and *p* < 0.01 vs. sh-NC.

**Figure 6:**
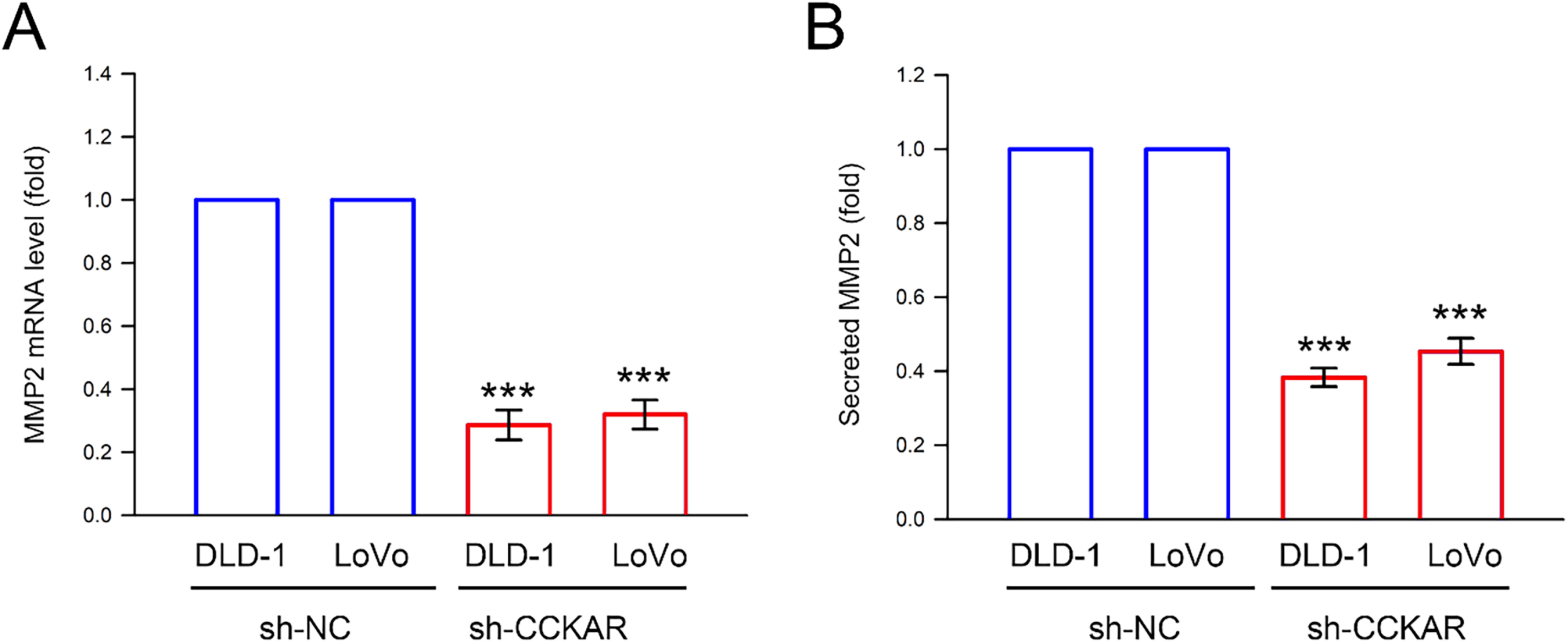
Knockdown of CCKAR reduces mRNA expression and protein secretion of MMP2 in human colon cancer cells. DLD-1 and LoVo cells were transfected with specific shRNA lentiviral constructs against CCKAR (sh-CCKAR) or control lentiviral constructs (sh-NC). The transfected cells were grown to 90% confluency, then (**A**) the cells were subjected to qPCR analysis to assess MMP2 mRNA expression level, and (**B**) the cultured medium was subjected to ELISA assay to determine secreted MMP2. GAPDH expression was used as internal control. ***, *p* < 0.001 vs. sh-NC.

In addition to the integrin/FAK axis, EMT and the Rho GTPase family are highly associated with the metastatic potential of colon cancer cells [26]. Therefore, the influence of CCKAR knockdown on expression of EMT markers, Rho GTPases, and uPA/uPAR was explored. As illustrated in Fig. 5A, knockdown of CCKAR led to an increase in the epithelial markers ZO-1 and E-cadherin, while simultaneously reducing the mesenchymal marker vimentin. Furthermore, protein levels of RhoA and CDC42 were decreased following CCKAR knockdown, whereas Rac1 levels remained largely unaffected (Fig. 5B). Given that uPA and uPAR are critical drivers of colon cancer invasiveness [27], their expression was also evaluated. Results in Fig. 5C demonstrate that CCKAR knockdown lowered uPA and uPAR protein levels in both DLD-1 and LoVo cells; however, levels of the uPA inhibitor, PAI-1, remained unchanged in both cell lines. The effects of CCKAR knockdown on gene transcription were also examined to complement the protein-level data. Fig. 5D–F demonstrate that CCKAR depletion significantly upregulated mRNA expression for ZO-1 and E-cadherin to 5.6-fold and 3.6-fold (DLD-1) and to 6.3-fold and 4.2-fold (LoVo), respectively; but downregulated vimentin, RhoA, CDC42, uPAR, and uPA to 56%, 72%, 68%, 38%, and 72% of control (DLD-1) and to 63%, 51%, 45%, 42%, and 64% of control (LoVo), respectively (*p* < 0.05). In contrast, no significant changes were observed in the mRNA levels of Rac1 and PAI-1. The effect of CCKAR knockdown on MMP2 production was the final element examined. As illustrated in Fig. 6, CCKAR knockdown significantly inhibited the expression and secretion of MMP2 at both the mRNA to 29% (DLD-1) 33% (LoVo) and protein levels to 37% (DLD-1) and 48% (LoVo) in average.

### 3.5 CCKAR Signaling Inhibition Modulates Gene Expression of Integrin **β**3/Paxillin, EMT Markers, Rho GTPases, uPAR/uPA, and MMP2 in Colon Cancer Cells

**Figure 7:**
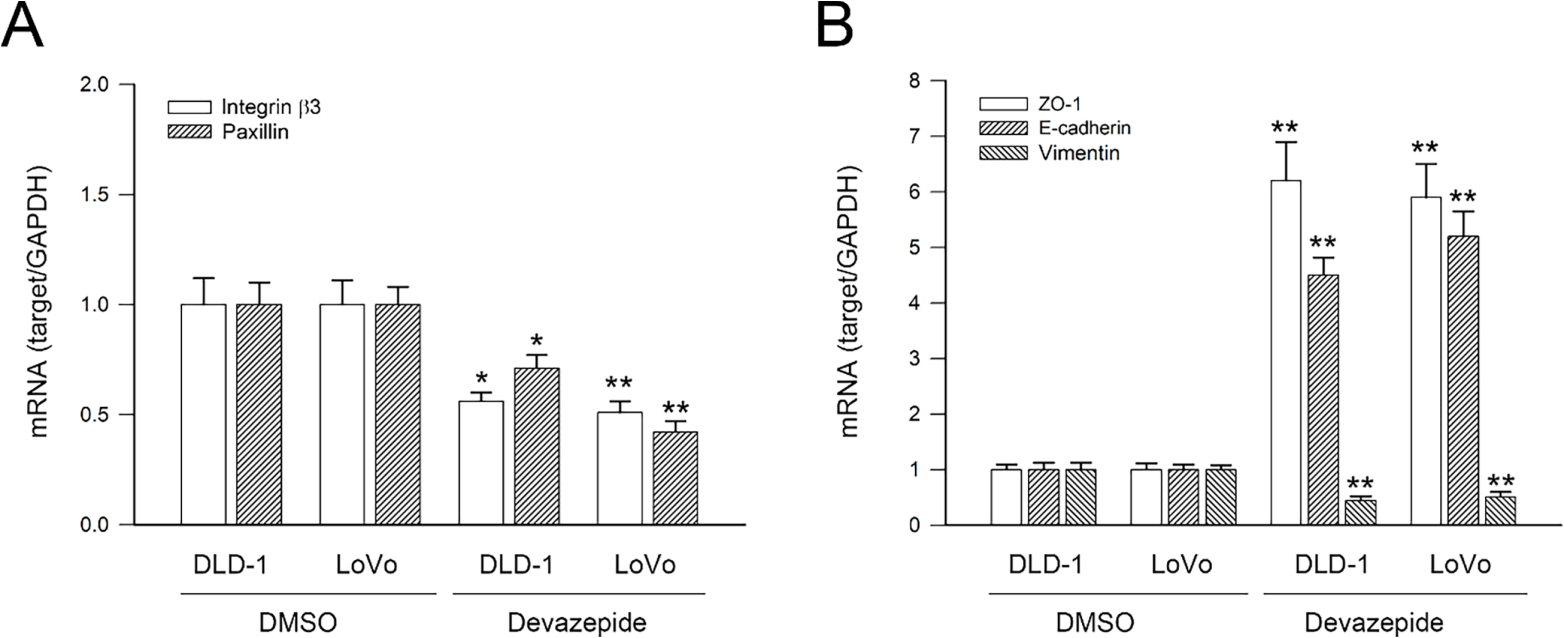

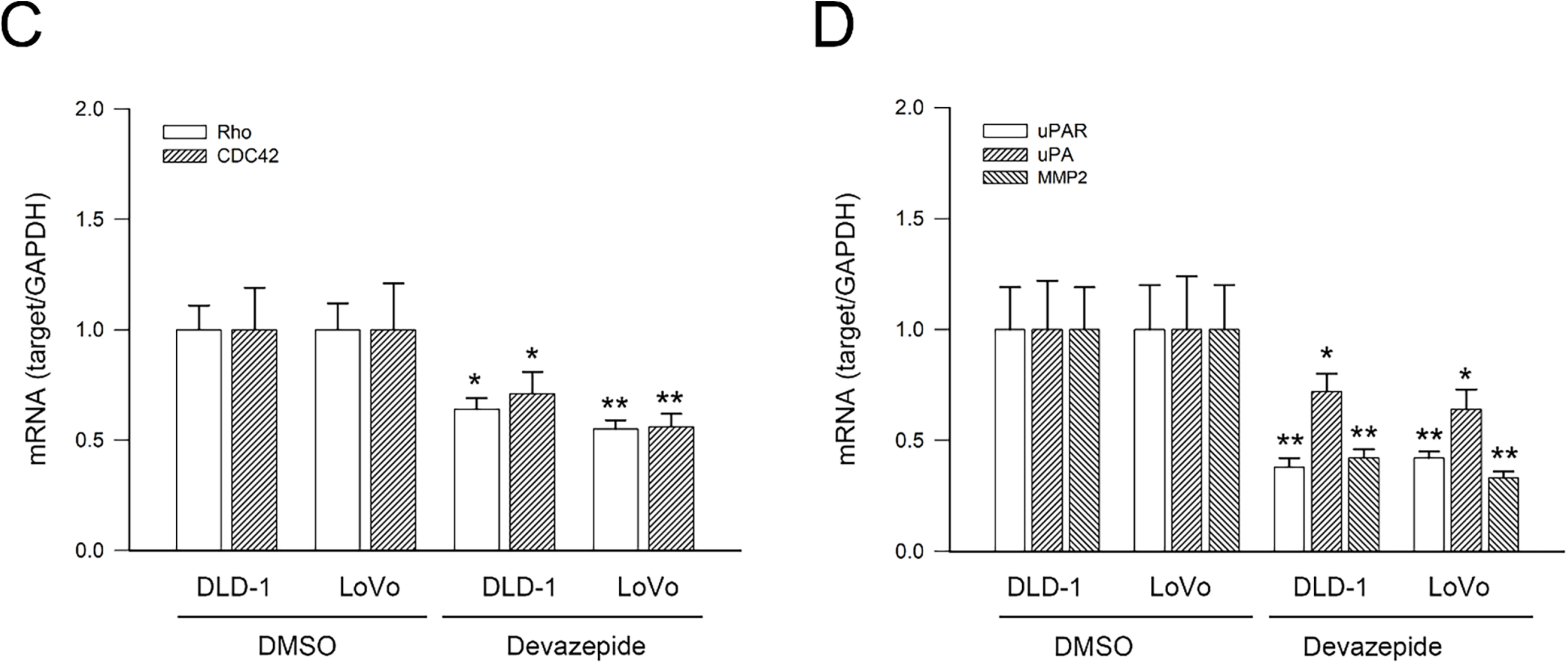
CCKAR signaling inhibition modulates mRNA expression of integrin β3/paxillin, EMT markers, Rho A/CDC42, and uPAR/uPA/MMP2 in human colon cancer cells. DLD-1 and LoVo cells were treated with devazepide (1 μM) for 6 h, and then subjected to qPCR analysis for mRNA expression of (**A**) Integrin β3 and paxillin, (**B**) ZO-1, E-cadherin, and vimentin, (**C**)Rho A and CDC42, and (**D**) uPAR, uPA, and MMP2. DMSO treatment was used as sham control. * and **, *p* < 0.05 and *p* < 0.01 vs. DMSO.

Finally, the role of CCKAR signaling in regulating integrin β3 and paxillin, EMT markers, Rho GTPases, uPAR, uPA, and MMP2 was evaluated. Consistent with findings from CCKAR-knockdown cells, treatment with the CCKAR antagonist devazepide significantly suppressed the mRNA expression of integrin β3, paxillin, vimentin, RhoA, CDC42, uPAR, uPA, and MMP2 to 0.68, 0.82, 0.44, 0.64, 0.71, 0.38, 0.72, and 0.42-fold of control (*p* < 0.05, DLD-1) and 0.45, 0.34, 0.51, 0.55, 0.56, 0.42, 0.64, and 0.33 (*p* < 0.05, LoVo), respectively. Meanwhile, devazepide enhancing the expression of ZO-1 and E-cadherin to 6.2 and 4.5-fold (*p* < 0.05, DLD-1, Fig. 7A,B) and 5.9 and 5.2-fold (*p* < 0.05, LoVo, Fig. 7C,D). These results suggest that CCKAR inhibition promotes an epithelial phenotype by upregulating E-cadherin and ZO-1 and downregulating mesenchymal and invasive markers, including vimentin, Rho A, CDC42, uPA, uPAR, and MMP2.

## 4 Discussion

The role of CCKAR in gastrointestinal physiology has been extensively studied, primarily in the context of pancreatic secretion, gallbladder contraction, and satiety regulation [28]. Accumulating evidence indicates that CCKAR plays a critical role in tumor progression and metastasis [9,29]. Consistently, our findings demonstrate that CCKAR is differentially expressed across human colon cancer cell lines, with notably higher levels in DLD-1 and LoVo cells. Functional assays revealed that silencing CCKAR markedly impaired cell motility and invasion in these cell lines. Mechanistically, CCKAR knockdown led to the downregulation of integrin β3, decreased phosphorylation of FAK, Src, and paxillin, and modulated expression of EMT markers, uPA and its receptor uPAR, as well as Rho family GTPases CDC42 and RhoA. These results suggest that CCKAR potentially promotes colon cancer cell invasiveness through coordinated regulation of integrin/FAK/Rho GTPase signaling and the uPA/uPAR axis.

Emerging evidence underscores the significance of integrin-FAK signaling in colorectal cancer metastasis. For instance, Integrin α6 and β4 are selectively packaged in tumor-derived exosomes and found to enhance lung metastasis of colon cancer by promoting vascular endothelial proliferation and tubulogenesis [30]. A comprehensive review further delineates the mechanistic contributions of integrin β4 to motility and EMT in tumors [31]. Moreover, high integrin β3 expression is reported to correlate positively with STAT3 signaling, and the STAT3/integrin axis promotes pancreatic cancer progression. Our findings demonstrate that CCKAR knockdown reduces integrin β3 expression and suppresses activation of the FAK/Src/Paxillin pathway, suggesting that CCKAR promotes colon cancer cell invasiveness through modulation of integrin β3 and its downstream signaling cascade. Notably, integrin αVβ5 was only marginally affected by CCKAR silencing, and whether CCKAR also regulates other integrins remains to be clarified.

EMT is a well-recognized mechanism of colon cancer progression and metastasis [32]. ZO-1 is a characteristic factor of tight junctions, which has also been demonstrated in E-cadherin junctions [33,34]. Decreased ZO-1 expression has been identified to be associated with increased invasiveness in liver cancer [35] and breast cancer [36], and colorectal cancer [37]. Among mesenchymal markers, vimentin serves as a key mediator that integrates EMT processes with colorectal cancer progression and the development of resistance to histone deacetylase inhibitors [38]. Our findings unveil that CCKAR knockdown suppressed EMT—characterized by the upregulation of ZO-1 and E-cadherin and downregulation of vimentin—thereby inhibiting the cellular changes required for motility and invasion. These findings depict that CCKAR may contribute to EMT activation in colon cancer cells.

Rho GTPase activities are intimately connected to the aggressive behaviors of cancer cells [39]. Notably, CCKAR knockdown downregulated Rho GTPases RhoA and CDC42, which are central regulators of actin cytoskeleton dynamics and cell polarity, underscoring the multifaceted role of CCKAR in promoting cellular invasiveness. Interestingly, CCKAR knockdown did not alter Rac1 protein levels in colon cancer cells. Prior literature indicates that Rho GTPases are regulated by specialized guanine nucleotide exchange factors (GEFs) [40]. This implies that CCKAR might trigger specific GEFs, like p115 RhoGEF, which exhibit selectivity for RhoA and CDC42 [41]. Nevertheless, the precise regulatory mechanisms governing CCKAR-mediated Rho GTPase expression require further investigation. The findings show that CCKAR contributes to regulating the protein level of Rho GTPases; however, whether CCKAR also regulates other Rho GTPases needs further investigation.

The breakdown of the extracellular matrix (ECM) is essential for tumor invasion. The uPA/uPAR system plays a key role in facilitating this ECM degradation, thereby promoting cell invasion. Our results revealed that CCKAR knockdown reduced both uPA and uPAR expression, suggesting that CCKAR is involved in modulating the uPA/uPAR system. In addition to uPA/uPAR, extensive literature has established associations between MMP2 aberrant expression and colon cancer evolution, demonstrating correlations with key clinicopathological parameters including staging, metastatic status, and overall survival [42]. Our findings reveal that CCKAR knockdown downregulates mRNA expression and secretion of MMP2, indicating that CCKAR is involved in the regulation of MMP2 in colon cancer cells.

Several selective and non-selective CCK receptor antagonists have been developed and characterized, including the CCKAR antagonists devazepide and lorglumide, as well as the non-selective antagonist proglumide. Previous *in vitro* studies have shown that blockade of CCKAR by devazepide and lorglumide,can reduce proliferation of colon cancer cell [43], induce apoptosis of Ewing tumor cells [44], and growth and invasion of pancreatic cancer cell [45], suggesting that CCKAR represents a pharmacologically tractable target for cancer treatments. CCKAR is also annotated as a druggable receptor in chemical databases such as ChEMBL (Target ID: CHEMBL2871), PubChem, and DrugBank, where several ligands and bioactivity entries are reported. However, to date, there are no clinically validated anticancer compounds specifically targeting CCKAR. Our results indicate that devazepide treatment significantly modulates integrin β3/paxillin, EMT markers, RhoA/CDC42, uPAR/uPA, and MMP2 expression, suggesting that CCKAR inhibition may have anti-invasive potential for colon cancer cells. These findings indicate that while CCKAR exhibits characteristics consistent with a potential therapeutic target, further studies are required to optimize selective inhibitors and assess their antitumor efficacy *in vivo*.

Despite the significant findings presented here, our study is subject to several limitations that should be considered when interpreting the results. First, while we screened six colon cancer cell lines for CCKAR expression, our functional knockdown experiments were conducted using only two cell lines (DLD-1 and LoVo). While these models provided consistent results, additional cell lines or primary patient-derived cells would further enhance the generalizability of our findings across the heterogeneous landscape of colorectal cancer. Second, this study relied on shRNA-mediated gene silencing, which may leave residual protein levels that could influence cellular behavior. Furthermore, our observations were strictly *in vitro*; therefore, they lack the complexity of the *in vivo* tumor microenvironment, such as interactions with stromal and immune cells or systemic physiological factors. Future research utilizing murine xenograft models or organoid cultures is necessary to validate the role of CCKAR in tumor metastasis in a more biologically relevant context.

## 5 Conclusion

In conclusion, our findings suggest that CCKAR promotes colon cancer cell motility and invasion via the integrin/FAK/Rho GTPase pathway and uPA/uPAR axis, potentially alongside EMT activation. CCKAR is a potentially druggable receptor that warrants further investigation as a therapeutic target in colon cancer. Comprehensive *in vivo* validation, clinical association analyses, and studies employing selective CCKAR antagonists will be necessary to substantiate its therapeutic relevance and to identify effective approaches for blocking its pro-invasive signaling.

## Supplementary Materials



## Data Availability

All data supporting the findings of this study are available within the paper and in the Supporting Information. Additional details can be provided by the corresponding authors upon reasonable request.

## Supplementary Materials

The supplementary material is available online at https://www.techscience.com/doi/10.32604/or.2026.074231/s1.
