# Supplementary material for: Cholecystokinin A Receptor Knockdown Diminishes Colon Cancer Cell Invasive Potential via Modulation of Integrin/FAK, EMT, and uPA/uPAR/MMP2 Axis
Source: Oncol Res. 2026 Mar 23;34(4):22. doi: 10.32604/or.2026.074231 (PMC13040287; doi:10.32604/or.2026.074231)
Supplement: Supplementary file 1 [file OncolRes-34-74231-s001.docx]

**Supplementary data**

**Table S1.** Information for antibodies used in Western blots

| **Target** | **Dilution (fold)** | **Cat. No.** | **Manufacturer** |
| --- | --- | --- | --- |
| CCKAR | 1000 | ab75153 | abcam |
| β-actin | 2000 | sc-8432 | Santa Cruz Biotechnology |
| integrin αV | 1500 | #4711 | Cell Signaling |
| integrin β3 | 1500 | #4702 | Cell Signaling |
| Focal adhesion kinase (FAK) | 1500 | sc-418 | Santa Cruz Biotechnology |
| pY397-FAK | 1500 | sc-271126 | Santa Cruz Biotechnology |
| pY925-FAK | 1500 | #3284 | Cell Signaling |
| Src | 1500 | #2108 | Cell Signaling |
| pY416-Src | 1000 | #2101 | Cell Signaling |
| paxillin | 1500 | #2542 | Cell Signaling |
| pS178-paxillin | 1000 | ab277786 | abcam |
| pY118-paxillin | 1000 | #2541 | Cell Signaling |
| ZO-1 | 1500 | #8193 | Cell Signaling |
| E-cadherin | 1000 | #3195 | Cell Signaling |
| vimentin | 1500 | #5741 | Cell Signaling |
| RhoA | 1000 | sc-418 | Santa Cruz Biotechnology |
| CDC42 | 1000 | #2466 | Cell Signaling |
| Rac1 | 1000 | #2465 | Cell Signaling |
| urokinase-type plasminogen activator (uPA) | 1500 | #15800 | Cell Signaling |
| urokinase-type plasminogen activator receptor (uPAR) | 1000 | #12863 | Cell Signaling |
| plasminogen activator inhibitor-1 (PAI-1) | 1500 | #11907 | Cell Signaling |
| goat anti-mouse IgG-HRP | 2000 | sc-2005 | Santa Cruz Biotechnology |
| goat anti-rabbit IgG-HRP | 2000 | sc-2004 | Santa Cruz Biotechnology |

abcam (Cambridge, UK); Santa Cruz Biotechnology (Santa Cruz, CA, USA); Cell Signaling (Danvers, MA, USA)


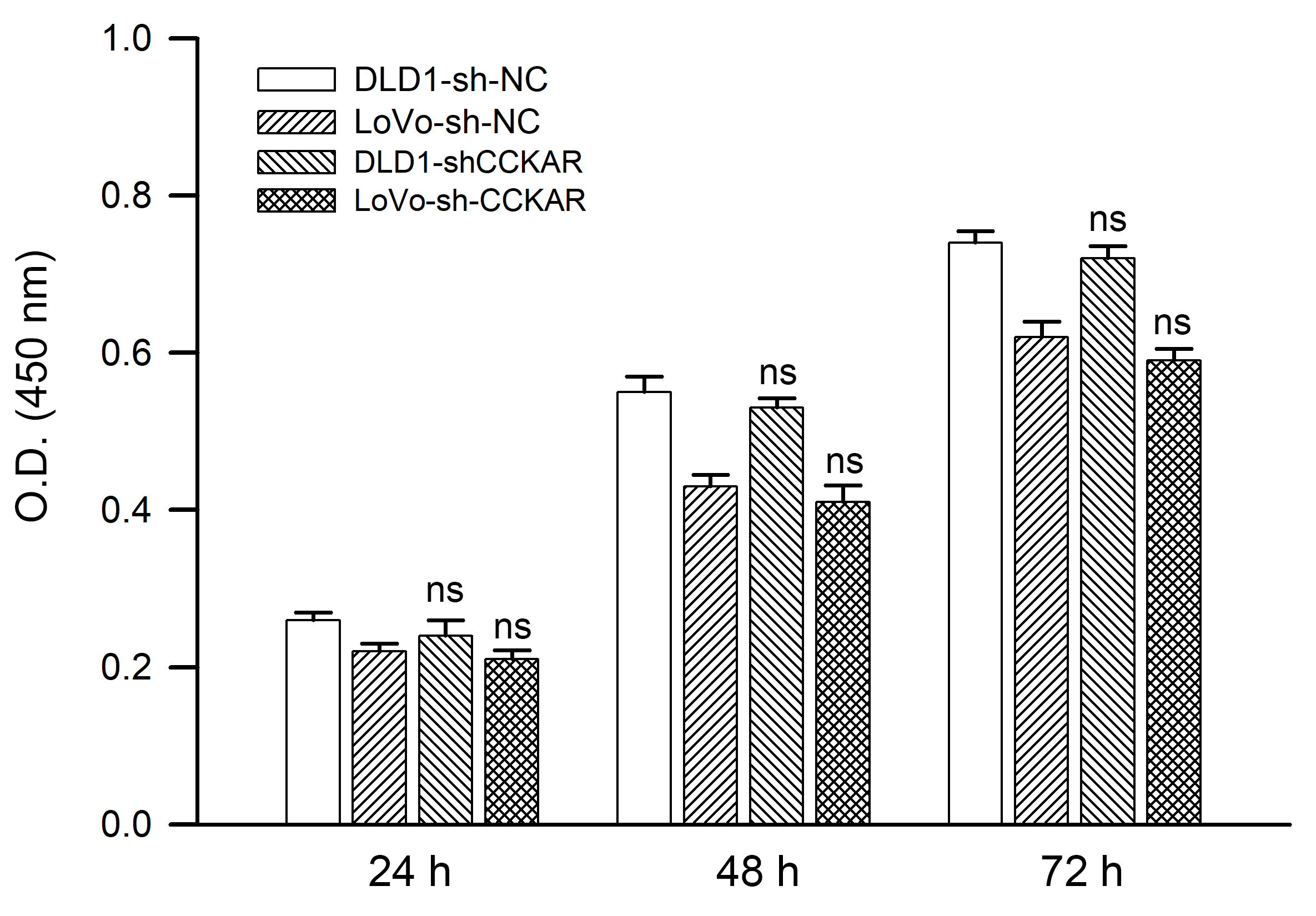


**Figure S1. Effects of CCKAR knockdown on cell proliferation of colon cancer cells.** Cells were transfected with sh-NC or sh-CCKAR, cultured for 24 h, 48 h, or 72 h, and then subjected to cell proliferation assessment using CCK-8 assay. ns, no significant changes between sh-NC and sh-CCKAR groups.
